# Unique Athletic Gut Microbiomes and Their Role in Sports Performance: A Narrative Review

**DOI:** 10.5114/jhk/202642

**Published:** 2025-10-01

**Authors:** Kinga Humińska-Lisowska, Paweł P. Łabaj, Kinga Zielińska

**Affiliations:** 1 Faculty of Physical Culture, Gdansk University of Physical Education and Sport, Gdansk, Poland.; 2 Malopolska Centre of Biotechnology, Jagiellonian University, Krakow, Poland.

**Keywords:** gut microbiome, elite athletes, training adaptation, probiotics, gut health

## Abstract

The human gut microbiome, a diverse community of microorganisms, plays a crucial role in digestion, metabolism, immune function, and brain health. Key metabolites produced by the gut microbiota, such as short-chain fatty acids (SCFAs) and bile acids, are essential for energy production, metabolic regulation, and immune system modulation. The gut microbiome's composition is influenced by factors including diet, exercise, sleep, and age, and disruptions are linked to various health conditions. Elite athletes exhibit unique gut microbiota profiles that contribute to their exceptional performance and recovery. Their microbiomes are not only richer, but also possess unique microorganisms and functional capabilities, alongside distinct genetic landscapes that support their high-level physiological demands. This review focuses specifically on the athletic gut microbiome, exploring how it differs from that of an active or a sedentary individual, adapts to different training phases, extreme conditions like heat and hypoxia, and prolonged exertion. It highlights the dual role of the gut microbiome in both enhancing athletic performance and potentially contributing to disease development, particularly due to the prolonged exertion and stress associated with years of intense competition. The review also explores the implications of microbiome changes following periods of intense physical activity and their impact on the athlete's overall health. Finally, it evaluates athlete-specific interventions, including prebiotics, probiotics, and synbiotics, aimed at mitigating negative effects on the gut microbiome while supporting health and optimizing performance.

## Introduction

The human gut microbiome is a community of microorganisms, including bacteria, viruses, and archaea, that interact with each other and with their host in a specific ecological niche. The microbiome is a dynamic community, displaying a wide range of functions and producing various metabolites (Berg et al., 2020). As early as in 2010, it was termed “the second genome of the human body” due to its crucial role in digestion, metabolism, immune function, and even brain health (Liu et al., 2022; Zhu et al., 2010).

Key metabolites produced by gut microbiota include short-chain fatty acids (SCFAs) like acetate, propionate, and butyrate, which are derived from the fermentation of dietary fibres and serve as an energy source for colonocytes, regulate immune function, and maintain gut barrier integrity. Additionally, bile acids assist in fat digestion and regulate lipid metabolism, while vitamins K, B12, biotin, and folate are essential for blood clotting, energy metabolism, and DNA synthesis. Neurotransmitters such as serotonin and GABA influence brain function and mood regulation through the gut-brain axis, and tryptophan metabolites modulate immune responses and maintain gut homeostasis.

The human gut microbiome is influenced by various host factors, including sleep (Smith et al., 2019), diet (Asnicar et al., 2021), exercise (Clauss et al., 2021) and age (Ghosh et al., 2022), along with other environmental determinants such as the host genome, stress, illness, and drugs (Rogers and Aronoff, 2016). Disruptions in the gut are linked to various conditions, including obesity, diabetes, inflammatory bowel disease, and neurological disorders (Kinross et al., 2011).

Due to the links of the gut microbiome with health, and its ability to adapt to various environmental conditions, it has been extensively studied in the context of athletic performance (Mohr et al., 2020). Elite athletes exhibit extraordinary physiological and metabolic adaptations, including enhanced muscular strength, aerobic capacity, energy expenditure, and thermoregulation, offering valuable insights into gut microbiome research. The unique gut microbiota that athletes possess contributes significantly to athletes’ health, well-being, and performance by harvesting energy, modulating immunity, and impacting mucosal and brain health. It indirectly affects exercise performance, recovery, and illness patterns through mechanisms like myokine signalling, cytokine modulation, hypothalamic-pituitary-adrenal axis activation, and metabolic pathways. Understanding these interactions is crucial for athletes aiming to enhance performance and recovery, and it also broadens our comprehension of microbial impacts on human health and elite-level performance.

Elite athlete status is not universally defined (Ds et al., 2013). However, the most widespread definition is competing at the highest national or international level and adherence to a rigorous training regimen (Guilherme et al., 2022; Ii and Va, 2009; Rankinen et al., 2016). Therefore, we focused our analysis on individuals who were defined as elite in the original publications, and who consistently competed at the highest level. In this review, we examined the gut microbiome in the context of elite athletic performance, focusing on its distinct characteristics and the unique mechanisms and environments that influence it. We began by outlining the uniqueness of the athlete’s gut microbiome, highlighting the specific features that differentiate it from the general population. Then, we explored how elite athletes had a distinct microbial composition and diversity that contributed to their enhanced metabolic and physiological functions. Building on this foundation, we delved into sport-specific and training-induced adaptations in the athletic gut microbiome, exploring how different types of sports, such as endurance, strength, and team sports, and different training intensities uniquely shaped microbial communities and their functional capacities. We discussed the mechanisms by which specific training regimens influenced gut microbiota composition, highlighting the interplay between exercise modality, duration, and frequency on microbial diversity and metabolic performance.

Next, we explored the dynamic changes that occurred in response to various training phases and interventions, demonstrating the remarkable adaptability of athletes striving for peak performance. We then discussed how adaptation to extreme conditions, such as heat, cold, altitude, and travel, affect the athlete’s gut microbiome. We examined the specific changes in microbial composition and function induced by these environmental stressors and how these adaptations supported athletes' performance and health under such challenging conditions.

Additionally, we highlighted the dual role of the gut microbiome in both enhancing performance and potentially contributing to disease development, particularly due to the prolonged exertion and stress associated with years of intense competition. We examined the implications of microbiome changes following periods of intense physical activity and their impact on the athlete's overall health. Understanding these risks is essential for developing strategies to maintain a healthy gut microbiome, thereby supporting long-term athletic performance and well-being.

Finally, we reviewed specific interventions for athletes aimed at improving health, performance and recovery. We discussed the use of prebiotics, probiotics, and synbiotics tailored for athletes and how these interventions can modulate the gut microbiome to support optimal performance and mitigate negative effects associated with intense training. By synthesizing current research on these topics, our review aimed to provide valuable insights for athletes, coaches, and healthcare professionals seeking to optimize performance and health through targeted microbiome interventions.

## Microbial Metabolic Pathways and Their Molecular Impact on Athletic Physiology

The influence of the gut microbiota on athletic performance is multifaced, involving various metabolic pathways, immune responses, and neurochemical changes. Understanding these mechanisms may provide insight into how athletes can optimize performance and recovery.

One of the primary ways in which the gut microbiota affects athletic performance is by modulating metabolic pathways. Gut bacteria are crucial in the fermentation of dietary fiber, resulting in the production of SCFAs which serve multiple functions. Acetate, propionate, and butyrate each play separate but complementary roles in metabolic processes. Acetate can be absorbed by peripheral tissues and utilized as a substrate through the tricarboxylic acid (TCA) cycle for energy production (Besten et al., 2013). It also has an influence on cholesterol metabolism and lipogenesis in the liver, optimizing lipid levels and energy storage, which may be beneficial for athletes (Perry et al., 2016). In addition, acetate plays a role in regulating appetite and energy intake, potentially helping athletes maintain optimal body composition and energy levels (Frost et al., 2014). Propionate influences glucose metabolism by acting on the liver and muscles as an energy supply, potentially improving endurance performance (Petersen et al., 2017). In addition, certain bacteria, such as *Veillonella spp*., can metabolize lactate produced during intense exercise into propionate, improving endurance and recovery (Scheiman et al., 2019). On the other hand, butyrate is particularly important for colonocytes, as it promotes a healthy interstitial barrier and reduces inflammation (Clarke et al., 2014). Together, acetate, propionate, and butyrate help maintain energy balance, metabolic health, and immune function, which are crucial for high-performance athletes.

The gut microbiota also plays a critical role in the modulation of the immune system, which is essential for athletes who often face physical stress and potential exposure to pathogens. SCFAs such as butyrate have anti-inflammatory properties and can enhance the production of regulatory T cells which help maintain immune homeostasis and prevent chronic inflammation. Furthermore, a healthy gut microbiome supports the integrity of the intestinal barrier, preventing the translocation of pathogens and toxins that can trigger immune responses and systemic inflammation (Takiishi et al., 2017). Interestingly, Gosiewski et al. (2017) observed that bacterial DNA can be also detected in the blood of healthy people, suggesting that bacteria can translocate into the bloodstream without necessarily causing a pathological state (Gosiewski et al., 2017). This finding highlights the dynamic interaction between the gut microbiome and the host immune system, even under normal physiological conditions.

The gut-brain axis is a bidirectional communication system between the gut microbiota and the brain, influencing brain function and behaviour. This communication occurs through neural, hormonal, and immune pathways and plays a critical role in maintaining homeostasis and overall health. Recent research has highlighted the significant influence of the gut microbiota on brain function, with implications for mental health, motivation and stress resilience—factors that are particularly relevant to athletes. Gut bacteria are involved in the synthesis of neurotransmitters such as dopamine, norepinephrine, serotonin (plays a key role in mood regulation and mental well-being) (Cryan et al., 2019) and GABA (reduces neuronal excitability and promotes relaxation and stress reduction), which all together play a role in mood regulation, stress response, and cognitive function (Dicks, 2022). The modulation of these neurotransmitters by gut bacteria suggests a direct link between gut health and mental health, highlighting the potential for interventions that target gut microbiota to alleviate symptoms of anxiety and depression in athletes, thereby enhancing their psychological resilience and performance.

Recent research has shown that gut microbiota can affect the central nervous system (CNS) through several mechanisms, including metabolite production, modulation of the immune system, and direct neural pathways via the vagus nerve (Cryan et al., 2019). Exercise-induced neurochemical changes are also modulated by gut-derived metabolites. Research has shown that gut metabolites, such as endocannabinoid-like compounds, can stimulate sensory neurons that increase dopamine levels (referred to as the pleasure neurotransmitter) in the brain during exercise. This neurochemical pathway has been associated with increased motivation and exercise performance by promoting reward-related behaviour and reducing the perception of effort (Dohnalová et al., 2022). Furthermore, the gut microbiota modulates the hypothalamic-pituitary-adrenal (HPA) axis, which controls the body's stress response (Farzi et al., 2018). Dysbiosis, or an imbalance in the gut microbiota, can lead to an exaggerated stress response and increased levels of cortisol which is a stress hormone (Rusch et al., 2023). Conversely, a healthy and balanced gut microbiome can help regulate the HPA axis, reducing stress and improving overall resilience. This regulation is crucial for athletes who are often exposed to physical and psychological stressors.

Emerging evidence also suggests that the gut-brain axis can influence cognitive function, which is critical for strategic thinking, decision-making, and reaction times in sports. For example, SCFAs like butyrate have neuroprotective effects and can enhance the brain-derived neurotrophic factor (*BDNF*) expression, promoting neurogenesis, executive function and other cognitive processes. By supporting gut health, athletes may be able to improve their cognitive performance and recovery from mental fatigue.

Research has shown that exercise can alter the composition of the gut microbiota, which in turn affects the production of neurotransmitters and neurotrophic factors, further influencing brain function and mental health (Bested et al., 2013). Integrating these mechanistic insights may help understand how specific gut bacteria and their metabolites contribute to the overall health and performance of athletes.

## The Uniqueness of the Athletic Gut Microbiome

Moderate physical activity is not sufficient to promote strong, athletic-like shifts in the human gut microbiome, and it is the extended period of intense training that grants the unique athletic status. Petri et al. (2024) compared athletes to healthy individuals with three levels of activity: high, moderate and none. Similarly to other studies, they identified an enrichment of butyrate-producing species and higher butyrate concentrations in elite soccer players than in the other groups. Interestingly, genus *Phascolarctobacterium* and species *Phascolarctobacterium succinatutens*, pro- inflamatory inhibitor and propionate producer, appeared to be associated only with higher levels of physical activity. Notably, higher levels of activity were probably needed to promote an increase in this gut microbiota species. These findings underscore the distinctive gut microbiota of athletes, highlighting the significant impact of high levels of physical activity on microbial composition and metabolic output.

In the early 2010s, studies began to show that elite athletes had a more diverse and richer gut microbiome than non-athletes (Clarke et al., 2014). This diversity was linked to improved metabolic health, enhanced immunity, and better overall performance. Landmark studies, such as those by Clarke et al. (2014) and Petersen et al. (2017), demonstrated that exercise can significantly alter the composition of the gut microbiota, leading to the abundance of beneficial bacteria that produce short-chain fatty acids (SCFAs) and other health-promoting metabolites. A recent comparison of esports players, endurance athletes and students by Kulecka et al. (2023) revealed that while esports players and students presented significant differences with respect to nine bacterial species and nine amino acids, the most distant group was that of endurance athletes. The marked impact of intense training on the gut microbial structure and function was evident in the form of 45 and 31 bacteria differentiating between athletes and esport players or students, respectively. In addition to that, athletes had significantly greater bacterial diversity, Firmicutes/Bacteroides ratios, enterotype profiles, metagenome functional contents and SCFA concentrations.

Findings combining metagenomics and metabolomics provide further evidence for the unique adaptation of the athletic gut microbiome to increased metabolic and physiological demands of physical activity. Fontana et al. (2023) found a major athlete-specific enzymatic cluster, related to as many as 752 enzymes and 73 high biological impact synthases (Fontana et al., 2023). The cluster was complemented by a recurrent pattern defined by SCFA microbial producers such as *Faecalibacterium, Eubacterium, Blautia* and *Ruminococcus*. Similarly, Wosinska et al. (2024) examined the athletic gut microbiome at a functional level through the most extensive collection of metagenome-assembled genomes (MAGs) from athletes recently compiled (Wosinska et al., 2024). The new dataset allowed for the identification of exercise-associated gene clusters, among which 19.743 showed positive and 9.296 negative associations. The authors went a step further to fully exploit the novel nature of the catalog and proposed a MAG recovery strategy to improve classification and identification of novel MAGs. As a result, 9 MAGs differentially abundant in the athlete population, belonging to genera relevant in the context of exercise (*Rumonococcus* and *Alistipes*), were discovered. Notably, athletes exhibited a high prevalence of genes associated with stress adaptation, muscle recovery, as well as vitamin and amino acid synthesis compared to the control group.

Due to increased energetic and nutritional needs for training and recovery, athletes tend to display distinct dietary behaviour than the rest of the population. Diet, in addition to high intensity exercise, appears to be a key player shaping the athletic gut microbiome. Clarke et al. (2014) noted that the total energy intake was significantly higher in athletes than in controls and found that protein accounted for considerably more (22%) of the total energy intake of athletes. Interestingly, dietary protein consumption was associated with the enhanced microbiota diversity in athletes which, in turn, positively correlated with creatine kinase (a marker of extreme exercise). Elevated creatine kinase levels in athletes, a result of intense exercise, could therefore be linked with increased protein intake. This finding was suggestive of an impact of physical activity on the use of dietary nutrients by the microbiota of the gut. In a continuation study, Barton et al. (2018) compared athletes and controls at the functional metabolic level to identify diet-influenced pathways specific to highly trained individuals. They observed that athletes had enriched pathways related to amino acid, antibiotic biosynthesis and carbohydrate metabolism, as well as faecal metabolites (SCFAs in particular). While all those were associated with enhanced muscle turnover (fitness), propionate was strongly correlated with protein intake and butyrate with dietary fibre. These provided strong evidence for the characteristic dietary contributions of athletes to the unique functional composition of the enteric microbial system, so distinct from the rest of the population.

The athletic gut microbiome appears to be a rich source of antimicrobial peptide-producing bacteria (Wosinska et al., 2022). Wosinska et al. (2022) identified a broad range of potential bacteriocin classes present in the athlete gut, suggesting a potential role of the associated species as an alternative to widely used antibiotics. The in-vitro approaches resulted in the generation of 284 gut isolates which, astonishingly, demonstrated inhibitory activity against *Clostridioides difficile* and/or *Fusobacterium nucleatum*. This suggests that the unique microbiome composition of athletes may serve as an alternative to widely used antibiotics, offering new avenues for antimicrobial therapies.

Remarkably, alterations in the athlete's gut microbiome might even differently modulate their motivation for exercise. While the recent study by Dohnalova et al. (2022) was performed on mice, the groundbreaking results highlight the importance of the gut metabolites triggering exercise-induced neurochemical changes in the brain that could be similar in humans. The research found that endocannabinoid metabolites in the gut stimulated the activity of the TRPV1-expressing sensory neurons, and therefore elevated dopamine levels in the brain during exercise. Strikingly, the stimulation of this pathway improved running performance, while microbiome depletion or mechanism blockage appeared to abrogate exercise capacity. The results indicated that the gut-derived signals could enhance motivation for exercise. More importantly, they provided a microbiome-dependent explanation for interindividual variability in exercise performance. These findings underscore the unique interplay between gut metabolites and brain function in athletes, revealing how the microbiome can influence motivation and determine top-level performance. Genetic studies further support the importance of neurotransmitters such as dopamine or serotonin in exercise motivation and performance (Humińska-Lisowska, 2024).

However, differences between athletes and nonathletic populations are not confined to the bacterial part of gut microbiota. Limited research focused on archeons revealed an enrichment of *Methanobrevibacter smithii* transcripts in a number of professional versus amateur cyclists (Petersen et al., 2017). This archeon, displaying upregulation of genes involved in the production of methane, was associated with similar upregulation of energy and carbohydrate metabolism pathways. Potentially greater uniqueness of the athlete gut microbiome can be realized by expanding studies to include archaea and other microorganisms. This broader perspective could deepen our understanding of how diverse microbial communities contribute to athletic performance and health.

In summary, the athletic gut microbiome is distinguished by its complexity and adaptability. Physical activity alone does not induce the profound microbial shifts observed in athletes, highlighting the unique gut environment that emerges from extended periods of intense training and athlete-specific diet. Studies agree that athletes have more diverse microbiomes, enriched with beneficial bacteria that enhance metabolic functions and support overall performance. Interestingly, they might also experience distinct mechanisms of motivation for exercise. This uniqueness extends beyond bacteria to include other microorganisms, such as archaea, which may further contribute to the specialized gut ecosystem of athletes. However, our knowledge of the role of non-bacterial species in the athletic gut microbiome is insufficient, as studies restricted to athletic populations only are currently very limited.

## Sport-Specific and Training-Induced Adaptations in the Athletic Gut Microbiome

One of the first studies comparing the gut microbiome between different sports identified distinct microbiome and metabolome compositions in subgroups of elite Irish athletes (O’Donovan et al., 2020). Athletes participating in sports with a high dynamic component (race walking, field hockey) were the most distinct at the species level, while those involved in sports with high dynamic and static components (triathlon, rowing, boxing, cycling) were functionally dissimilar. Twenty-one metabolites, such as lactate, succinate and creatinine, were found to be significantly different between the groups. Importantly, no dietary differences were observed between groups. This finding not only again highlights the uniqueness of the athletic gut microbiota resulting from increased physical activity, but also from the characteristics of the practised sport.

There is compelling evidence that genetic factors influence several phenotype traits related to physical performance. It is estimated that even up to 66% of the variance in athletic status could be attributed to genetic factors (Bıçakçı et al., 2024; Eynon et al., 2011; Moor et al., 2007). Diaz et al. (2020) were first to investigate the role of the *GALNTL6* gene, known to be 1.23 times more prevalent in the endurance athletic versus control population in the C allele version, in relation to physical performance. Their groundbreaking discovery revealed that, unexpectedly, the T allele version was associated with favourable anaerobic performance and overrepresented in strength athletes. Similarly, a follow-up study by Zmijewski et al. (2021) provided further associations of the T allele of the *GALNTL6* rs558129 polymorphism with the short distance swimming athlete status. Díaz Ramírez et al. (2020) proposed a new possible functional role of the *GALNTL6* rs558129 polymorphism with regard to short-chain fatty acid regulation and their anti-inflammatory and resynthesis functions in the gut microbiome. Thus, the results imply that the unique combination of genes and the associated gut microbiome profiles could allow for exceptional performances in different sport modalities (Díaz Ramírez et al., 2020).

While most studies focus on cross-sectional comparison of athletes and controls, there appears to be a substantial amount of gut microbiome variability across the training phases (Akazawa et al., 2023). Akazawa et al. (2023) observed fascinating differences between Japanese athletes in their transition and preparation periods, specifically higher abundance of *Bifidobacterium, Parabacteroides* and *Alistipes* and lower abundance of *Prevotella* in the preparation versus transition periods. What is more, a longitudinal study of short-track speed skaters revealed major shifts in the levels of *Bacteroides, Blautia* and *Bifidobacterium* across different training periods. The alterations were significantly correlated with changes in aerobic capacity and often with anaerobic power. Overall, the results indicated a potential contribution of the unique intestinal environment to athletic status.

Training periodization is often accompanied by dietary manipulation, ranging from extremely high-protein to high-carbohydrate diets. Furber et al. (2022) identified significant microbiome alterations specific to nutritional intake: high-carbohydrate diet improved time-trial performance and was associated with an increase in *Ruminococcus* and *Collinsella*, while high protein reduced performance and bacterial diversity (Furber et al., 2022). Interestingly, athletes who demonstrated the highest performance during dietary modifications exhibited fewer significant changes in microbiome community composition. This suggests that diet alterations associated with periodization can profoundly impact gut homeostasis—particularly affecting bacteriophages—and, in turn, influence endurance performance.

The gut microbiome of athletes exhibits a remarkable ability to respond rapidly to exercise, as demonstrated by multiple studies. Groundbreaking research by Scheiman et al. (2019) revealed increased abundance of *Veillonella atypica* in runners after completing the Boston marathon. Inoculation of this isolated strain into mice significantly increased exhaustive treadmill runtime, which was explained by *V.atypica* utilizing lactate as its sole carbon source into propionate, potentially enhancing athletic performance. Another study investigated the impact of probiotic *Veillonella atypica* FB0054 supplementation on anaerobic capacity and lactate concentration (Gross et al., 2024). While no changes in lactate concentration, hemodynamics nor bacterial community were observed, 14 metabolites were differentially expressed. Overall, supplementation with *V.atypica* maintained performance levels which tended to decline with a placebo.

In a recent study, distinct microbial alterations were observed after a maximal intensity aerobic intervention (Bruce test) in trained individuals stratified by VO_2max_ and anaerobic power (Humińska-Lisowska et al., 2024). Individuals with an athlete-level VO_2max_ experienced an increase in *Bifidobacterium longum*, a probiotic species commonly represented in commercial products, as well as a SCFA producer *Roseburia inulinivorans*, after the effort. In addition, correlations between maximal power output and SCFA producers *Eubacterium rectale, Blautia wexlerae* and *Intestinimonas timonensis* were observed. A subsequent study extended these findings by showing training-specific microbial shifts and their associations with serum biomarkers; notably, endurance-trained athletes exhibited correlations between butyrate- producing species (*Eubacterium rectale, Blautia wexlerae*) and muscle remodeling markers (TIMP-1, transferrin receptor, IL-15) following aerobic exercise, whereas strength-trained individuals demonstrated distinct microbial signatures linked to anaerobic adaptations (Humińska-Lisowska et al., 2025).

Remarkably, the athletic gut microbiome does not need weeks or even days to respond to a stimuli. Grosicki et al. (2019) observed considerable changes in a world-class ultramarathon runner’s gut as quickly as two hours after finishing the Western States Endurance Run. The most severe alterations included a nearly tripled Firmicutes/Bacteroides ratio, as well as a five-fold increase in the relative proportion of *Proteobacteria, Veillonella* (+14%) and *Streptococcus* (+438%), with a decrease in *Alloprevotella* (−79%) and *Subdoligranulum* (−0%). Those findings have greatly enhanced our understanding of the gut microbiome's role in athletic health and performance, as such rapid and pronounced shifts after a sport intervention had not been observed in microbiome studies before.

In summary, the athletic gut microbiome demonstrates a remarkable degree of specificity and adaptability, influenced not only by the general increase in physical activity, but also by the specific demands of different sports and training regimens. Existing studies highlight the dynamic nature of the microbiome in response to both sport-specific activities and the phases of training, revealing significant shifts in microbial composition, genetic landscape and metabolite production that correlate with athletic performance.

## Adapting to Extremes: How Heat, Cold, Altitude and Travel Impact the Athletic Gut Microbiome

Completing the Badwater Ultramarathon or the Ironman World Championships in Hawaii are examples of particularly demanding scenarios that push athletes to their physiological limits. Race-pace efforts in climates above 32 degrees Celsius are likely to trigger strong stress responses in the body, inhibiting the function of the immune and circulatory systems, and might even cause permanent damage to the nervous system (Geng et al., 2023). Ischemia in the gut is likely due to a severely reduced intestinal blood flow (Lambert, 2004). This, in turn, could have a substantial influence on the athletic microbiota—hypothetically in the form of an increased permeability of the intestinal mucosa and an expansion of pathogens (Lian et al., 2021).

While not many studies have explored this topic in detail, existing findings indicate relationships between epithelial injury markers and a number of bacterial families such as *Akkermansiaceae* and *Ruminococcaee* (Bennett et al., 2020). A study by Pugh et al. (1967) conducted during a running race in England in 1966 made a fascinating discovery that athletes were capable of minimizing the impacts of heat exposure (Pugh et al., 1967). Those authors found that successful marathon runners had sweat rates equal to the highest values seen in heat-acclimatized non-athletes and could tolerate exceptionally high rectal temperatures. Cinca-Morros and Alvarez-Herms (2024) concluded that athletes who maintained a healthy gut homeostasis could perform physical activity free of adverse heat-related consequences. This aligns well with the findings of Armstrong et al. (2018) who proposed that host factors, lifestyle and habitual dietary patterns could enhance efficient immune function (Armstrong et al., 2018). Thus, athletes might be better adapted to heat, demonstrating an impressive ability to mitigate the adverse effects of high temperatures on their bodies.

Comparable focus of attention has been given to the role of gut microbiota in cold environments. In this case, the role of microbiota as a key regulator of homeostasis is well established (Geng et al., 2023). Already, Wanner et al. (2014) observed that the exercise-increased core temperature of mice was more dependent on ambient temperature than on the exercise mode or intensity. The hypothesis that hyperthermia could be managed centrally led to subsequent appreciation of the importance of the gut microbiome in adaptation to low temperatures. Meng et al. (2020) made an unexpected discovery that exercise could reverse microbiome alterations attributed to cold exposure. Specifically, they observed that alpha diversity and the abundance of *Proteobacteria* would decrease in low temperatures but climb back up in response to exercise in the cold. What is more, they identified correlations between gut microbial communities and white fat browning, as well as observed increased protection against the negative cardiovascular effects of cold exposure. Interestingly, the effects were only evident when both cold and exercise were applied at once. This finding is particularly interesting from the elite training perspective, as athletes maintain their training regimes during winter and, depending on sport, might compete under harsh low-temperature conditions. The findings suggest novel mechanisms with additional health benefits attributable to both cold and exercise mediated via altered gut microbes, which might be specific to highly active throughout the year individuals such as athletes.

High or low temperatures are not the only extreme environment that athletes train and perform in. Another extreme case is high altitude training. Klessen et al. (2005) observed distinct gut microbiome composition related to altitude exposure above 5000 m in German mountaineers during their expedition to Nepalese Himalayas in 2002. The main changes included a decrease in commensal bacteria such as *Bifidobacteria*, with a simultaneous increase in the abundance of pathogens (*Enterobacteriaceae* such as *E. coli)*. This, in turn, was linked to a decreased immune function and an increased inflammatory response. A later study by Li et al. (2018) simulated altitude training to find major injuries in the small intestinal mucosa. Although their study was conducted on rats, it is very likely to explain mechanisms that lead to the loss of gut eubiosis in athletes training above 2000 m.

Training and racing in different climates are associated with substantial amounts of international travel. Donovan et al. (2020) analysed the gut microbiomes of Irish cricketers travelling in the lead to the 2016 World Cup. They found that travel, even at the level of a single timepoint (India), disrupted the normal gut microbiome balance. This led to temporary shifts in the gut microbial diversity and composition. Interestingly, the greatest fluctuations were observed in individuals who already experienced gastrointestinal distress at the previous travel timepoint and in whom different strains of *E. coli* were found. They also observed alterations in antibiotic resistance and virulence gene profiles, which could have detrimental effects on the wellbeing and performance of athletes. The findings are particularly intriguing for athletes who frequently engage in rigorous training and extensive travel. This combination of physical exertion and constant movement subjects them to significant stress, making them more vulnerable to gastrointestinal distress and other related symptoms.

In conclusion, athletes who train and compete in extreme environments, such as high temperatures, cold climates or high altitudes, face unique challenges that significantly impact their gut microbiomes. The body's stress responses to these conditions, including gut ischemia and altered microbial composition, can deteriorate athletic performance and overall health. Studies indicate that maintaining a healthy gut microbiome may help mitigate some of these adverse effects, underscoring the importance of understanding the gut's role in supporting athletes under such demanding conditions.

## Dark Sides of Athletic Performance: Indicators of Gut Dysbiosis and its Impact on Health

There is a fine line between a thriving gut microbiome supporting elite-level fitness and an excessive strain put on it, leading to underperformance. The very nature of intense athletic activity can compromise the integrity of the gastrointestinal lining (Crowson and McClave, 2020). During high-intensity exercise, the blood flow is redirected from the intestines to the working muscles, potentially resulting in splanchnic hypoperfusion and ischemia in the gut (Moses, 2005). Consequently, gastrointestinal discomfort limits performance during competitions and can lead to prolonged inflammation in the gut if not addressed with appropriate measures.

During the course of the 161-km Western States Endurance Run, 96% of competitors experienced at least one form of gastrointestinal discomfort (Stuempfle and Hoffman, 2015). Similar statistics were observed in long-distance triathletes. At some point in the race, 93% of participants experienced gut distress and almost 7% of them dropped out because of it (Jeukendrup et al., 1999). Prolonged intense exercise specific to athletes might cause a permanent dysbiotic state. Morishima et al. (2021) compared stool samples of female endurance runners and matched healthy controls to find differences in the gut microbiota, but also in the metabolites. Among all, they observed a higher concentration of succinate, an undesirable metabolite when accumulated in the lumen, in the athletes. This suggested that the microbiotas of runners were less eubiotic than those of controls.

A strong factor contributing to the chronic state of inflammation in the athletes’ guts is the consumption of highly processed sports nutrition, such as sports drinks and gels. Sometimes more commonplace than water in an athlete’s regimen, they can alter intestinal immune responses, as well as lead to enrichment of bacterial pro-inflammatory genes in the liver and disruption of faecal metabolites (Crowson and McClave, 2020). Furthermore, athletes are known to intake higher doses of pain-reducing non-steroidal anti-inflammatory (NSAIDs) and anti-asthmatic drugs, as well as antibiotics (Alaranta et al., 2008). NSAIDs interact with phospholipids and uncouple mitochondrial phosphorylation, leading to increased gut permeability and low-grade inflammation. The damage can also include erosions and ulcers, as well as bleeding, protein loss and perforation (Bjarnason et al., 2018). Antibiotics, on the other hand, can cause antibiotic-associated diarrhoea. Cumulative exposure to antibacterials during a lifetime leads to the depletion of commensal organisms, and eventually to the loss of microbiome biodiversity and severe illness (Crowson and McClave, 2020).

A healthy gut is known to have a positive regulatory effect on bone mass, hormonal regulation of the bone metabolism and production of metabolites that can act as messengers to bones (Crowson and McClave, 2020). Similarly, gut dysbiosis can disrupt bone formation via indirect stimulation or inhibition of osteoblasts and osteoclasts (Zhang et al., 2018). Ishizu et al. (2021) studied the effect of prebiotic food consumption on bone resorption in Japanese female athletes. Increased abundance of *Bifidobacterium spp*. as a result of the intervention highlights the role of gut microbiota in the bone metabolism, supporting the notion that a healthy gut can positively influence bone mass and metabolic regulation. Conversely, persistent gut dysbiosis, prevalent across intensely exercising athletes, can have a potentially detrimental effect on bone formation in the long term.

Chronic stress, such as exercise fatigue and to perform well at competitions, influences the hypothalamic-pituitary-adrenal (HPA) axis. When dysregulated, it can cause depression and anxiety (Crowson and McClave, 2020). Interestingly, the HPA axis is also the primary neuroendocrine pathway affected by gut microbes (Rusch et al., 2023). Fu et al. (2024) investigated the connection of pre-competition anxiety with gut microbiota and metabolites in wrestlers in different sports performances. They linked anxiety to *Oscillospiraceae UCG_005, Paraprevotella, Ruminococcaceae* and *TM7x*, as well as found that athletes who performed better had more diverse gut microbiomes. In addition to that, the researchers identified dissimilarities at the functional level: the most differentially enriched metabolites were linked to caffeine metabolism, lipopolysaccharide biosynthesis and VEGF/mTOR signalling pathways. Overall, different levels of pre-competition anxiety and correlated microbiome states provided strong evidence for the association between psychological and physiological indicators in athletes.

Wegierska et al. (2022) compiled a list of dysbiotic microbiota associated with inflammation, which could aid in the diagnosis of gut dysbiosis or overexertion of the athlete. *Anaerotruncus colihominis* is an anaerobic microbe that can cause bacteremia in immunocompromised individuals. *Bacteroides ovatus* is a primary trigger for systemic antibody responses in IBS. *Collinsella aerofaciens* ferments a wide range of carbohydrates, including starch, producing hydrogen and ethanol, which can increase intestinal gas levels when abundant. *Desulfovibrio piger* is a sulfur-reducing bacterium and its excessive presence is associated with IBD. *Dorea formicigenerans* is particularly common in individuals with nonalcoholic hepatic steatosis. *Escherichia fergusonii*, an opportunistic pathogen, is implicated in IBS. *Finegoldia magna* can cause bacteremia, visceral and skin lesions, and has been isolated in joint prosthesis infections. *Haemophilus influenzae* is responsible for serious infections in children. *Parabacteroides distasonis* can cause severe infections when combined with other bacteria. *Parabacteroides merdae* is mainly found in individuals with type 2 diabetes and IBS. *Peptostreptococcus anaerobius* can lead to systemic infections under immunosuppressed conditions, affecting various body regions such as the brain, the neck, the liver, and more. Finally, *Shigella boydii* is an opportunistic pathogen involved in inflammatory intestinal diseases. The presence or increased abundance of the aforementioned species can act as markers of the excessive strain on the athlete, and indicate compromised health and performance linked to the inflammation developing in the gut.

Remarkably, alterations in the gut microbiota are not limited to the tracking of the athletes’ guts. Soriano et al. (2022) took advantage of the gut-brain connection to suggest gut microbiota as a future biomarker for head injury diagnosis. Longitudinal investigation of college football athletes across a sports season revealed a decrease in the abundance for two bacterial species, *Eubacterium rectale* and *Anaerostipes hadrus*, after a diagnosed concussion. These findings underscore the potential of gut microbiota as a diagnostic tool for concussion and other head injuries.

In summary, athletes continuously strive for balance between maintaining a healthy gut microbiome and pushing it to the point of strain, which can lead to underperformance and long-term health issues. The demands of high-intensity exercise can compromise the integrity of the gut lining, leading to gastrointestinal discomfort, inflammation, and even permanent dysbiosis. Factors such as processed sports nutrition, frequent use of NSAIDs, antibiotics, and chronic stress further exacerbate gut health, potentially affecting bone metabolism, psychological well-being, and overall performance. Emerging research highlights the role of gut microbiota not only in managing these risks but also as a potential biomarker for overall health and even conditions such as head injuries, underscoring the intricate connection between gut health and athletic performance.

## Specialized Interventions for Athletes: Enhancing Health, Performance and Recovery

There are a number of dietary and pre/probiotic interventions that have already been proven to or could potentially influence the health and capacity of elite athletes (Paoli et al., 2024). These include interventions aiming to reduce training-associated fatigue and GI symptoms, improve adaptation and recovery, and enhance performance. In this review, we focus on the most interesting examples, and elite athletes specifically.

The number of prebiotic and synbiotic-oriented studies targeting athletic populations is very limited. Yet, there have been a few signals indicating the beneficial role of such interventions in athletic health and performance. For example, Kapoor et al. (2020) found that even a low daily dosage of a prebiotic partially hydrolyzed guar gum (PHGG) contributed to the maintenance of athletic gut health. Their study participants observed a reduction in diarrhoea and experienced gut microbiome shifts (significant alterations in *Bifidobacterium* and *Clostridium* subcluster XI). Zhang et al. (2018) compared the effects of synbiotic and prebiotic intake on the long-term health of male athletes. However, that time GOS, FOS, inulin, PDX, strawberry powder, and maltitol were used. The synbiotic group additionally received *L.casei, B.lactis* and *L.plantarum*. As a result of intaking synbiotics rather than prebiotics, athletes observed a decrease in the markers of inflammation, HR_max_, an enhanced lactic acid elimination rate and a lowered incidence of upper respiratory tract infections. Similarly, basketball players who took a multi-train probiotic (*Bifidobacteria* and *Lactobacilli*) in combination with 40 g of corn bran (dietary fibre) for 23 days experienced a decrease in the inflammatory process activity and peripheral blood lymphocyte apoptosis (Trushina et al., 2024). This suggested a positive influence of the intervention on the host immune status. Finally, a fascinating study by Quero et al. (2021) showed that athletes responded differently to synbiotics compared to sedentary individuals. A nutritional supplement containing probiotic strains (*Bifidobacterium lactis* CBP-001010, *Lactobacillus rhamnosus* CNCM I-4036, and *Bifidobacterium longum* ES1) as well as the prebiotic fructooligosaccharides improved physical activity, sleep quality and perceived general health and stress levels only in the athletes’ group. Furthermore, it increased dopamine solely in athletes, while increased systemic levels of IL-1beta and decreased corticotropin-releasing hormone in the sedentary group. Therefore, a response to the synbiotic appeared to be linked to an improved immunoneuroendocrine response in which all the above markers are involved, and which was dependent on the basal training status.

In terms of probiotics, athletes have been using them for a variety of reasons. A probiotic blend consisting of 5 strains: *Lactobacillus helveticus* Lafti L10, *Bifidobacterium animalis* ssp. lactis Lafti B94, *Enterococcus faecium* R0026, *Bifidobacterium longum* R0175 and *Bacillus subtilis* R0179, appeared to decrease the incidence of GI symptoms during training in elite cyclists (Schreiber et al., 2021). In addition, it decreased the mean rates of perceived exertion. The *Bifidobacterium longum subsp. longum* Olympic No. 1 probiotic significantly improved the results in a 12-min Cooper’s test and altered the overall abundance of the gut microbiota, specifically *Bifidobacterium* and *Lactobacillu*s (Lin et al., 2020). When Fukichi et al. (2022) gave a multi-strain lactic acid bacteria-fermented soymilk extract (LEX, containing *L.paracasei, L.brevis* and *L.plantarum*) to Japanese college endurance athletes, they observed different microbiome alterations as a result of a sports competition. The intake of LEX appeared to be associated with a significant reduction in yeast and fungal markers, neurotransmitters and mitochondrial metabolites. In a different study by Martarelli et al. (2011), *L.paracasei* in combination with *L.rhamnosus* demonstrated a strong antioxidant behaviour during a four-week training-intense period in a cycling team. Their results suggested that athletes who were particularly exposed to oxidative stress might benefit from the ability of such probiotics to increase antioxidant levels and neutralize the effects of reactive oxygen species. *Bifidobacterium lactis* W51, *Levilactobacillus brevis* W63, *Lactobacillus acidophilus* W22, *Bifidobacterium bifidum* W23, and *Lactococcus lactis* W58 in combination with vitamin D3 improved the epithelial cell permeability and extended time to exhaustion in mixed martial arts athletes (Przewłócka et al., 2023). After four weeks of supplementation, a significantly lower concentration of calprotectin was observed, and an augmentation of beta diversity within the probiotic + vitamin group. Those results were not noted in the vitamin-only group, suggesting the key role of bacterial strains in the beneficial effects within the probiotic + vitamin group. Finally, a combination of *Lactobacillus, Bifidobacterium* and *Streptococcus* significantly increased run time to fatigue in heat-exposed male runners (Shing et al., 2014). The main difference was observed in serum lipopolysaccharide concentration pre- and post-exercise, as well as a small reduction in gastrointestinal discomfort. While the multi-strain probiotic demonstrated a clear beneficial effect on performance under heat stress conditions, further research is necessary to explain the precise mechanisms underlying this effect.

While many probiotics are aimed at enhancing athletic performance, it is often compromised health that poses the greatest limitation. Therefore, athletes should prioritize mitigating the detrimental effects of prolonged intense exercise, which ultimately constrain their capacity to train and perform at an optimal level. A study investigating the effect of probiotic supplementation on the incidence of upper respiratory tract infections (URTIs) in athletes during three months of winter training found a strong health benefit, however, without performance benefits (Strasser et al., 2016). Athletes that did not take a multi-species probiotic (*Bifidobacterium bifidum* W23, *Bifidobacterium lactis* W51, *Enterococcus faecium* W54, *Lactobacillus acidophilus* W22, *Lactobacillus brevis* W63, and *Lactococcus lactis* W58) observed a significant decrease in post-exercise tryptophan levels and experienced a 2.2-fold increase in the incidence of URTI symptoms in comparison to those who took probiotics daily for 12 weeks. The ability of a different *Lactobacillus* strain, *L.fermentum* VRI-003 (PCC), to enhance the mucosal immune system of elite athletes was evaluated throughout a four-month winter training period in elite male distance runners (Cox et al., 2010). While, again, no substantial changes in running performance were observed, the probiotic intake was associated with a significant reduction in severity and length of respiratory illness. The results suggested that the maintenance of IFNgamma levels could be a potential mechanism promoting the beneficial outcomes. An earlier study observed a similar process, in which probiotics were even shown to enhance mucosal immunity in “overtraining” (Clancy et al., 2006). One month of daily *L.acidophilus* intake by fatigued athletes characterized by re-activation of Epstein Barr virus found that the IFNgamma secreted by T cells increased significantly to the levels observed in healthy athletes. This was the first study which provided evidence of a T cell defect in overtrained athletes and its reversal following probiotic therapy. Remarkably, the probiotics aiming to reduce symptoms of URTIs and gastrointestinal distress appeared to have different effects in males and females (West et al., 2011). While *L. fermentum* clearly increased in abundance and reduced the scale of gastrointestinal illness as the load increased in males, it did not experience a clear expansion and, strikingly, increased the load of lower respiratory symptoms in females. This was mirrored in the intake of cold and flu medications. Therefore, while *L.fermentum* was beneficial for training males, its ambiguities in the context of URTIs and gastrointestinal symptoms in females required further resolution.

While a range of interventions aiming to improve athlete health exists, there have been few attempts to use athlete-based observations to improve the health of the general population. One of the rare examples is a multi-strain Lactobacillus consortium developed by Fitbiomics (Bongiovanni et al., 2025). The probiotic, derived from elite athletes’ microbiomes, is reported to improve sleep quality, bowel movements and energy levels by 69%, 37% and 31%, respectively. In addition, the improvements are associated with a significant decrease in markers of oxidative stress. The findings highlight the untapped potential of customized probiotic treatments sourced from exceptionally fit individuals to enhance various aspects of health and performance in non-athletic people.

In summary, prebiotic, synbiotic and probiotic interventions can positively influence the health and performance of elite athletes. Studies on prebiotics and synbiotics, though limited, suggest benefits such as reduced gastrointestinal symptoms, enhanced recovery, and improved immune response. Probiotics, widely used by athletes, have demonstrated the ability to decrease gastrointestinal discomfort, enhance antioxidant activity, and support immune health, especially during intense training periods. Some probiotics, like multi-strain blends, have also been linked to improved endurance and reduced fatigue, though these effects can vary between male and female athletes (Barreto et al., 2024). Probiotics developed from athlete-derived microbiomes are being explored to benefit the general population, showing potential in improving sleep, energy levels, and gut health. While these interventions show promise, further research is needed to fully understand their mechanisms and optimize their use for both athletes and the broader public.

## Conclusions

In conclusion, athletes have a distinct and highly adaptable gut microbiome that is finely tuned for peak sports performance. Their microbiome exhibits functions not found in non-athletic individuals and responds to stimuli in unique ways. Due to these specialized characteristics, maintaining the health of an athlete's gut requires tailored considerations and interventions. While it may be unlikely for non-athletes to fully replicate the benefits of an athletic microbiome, the general population can still benefit from these insights by adopting athlete- mimicking health-promoting behaviours or utilizing athlete-derived probiotics.

The majority of existing studies rely on 16S rRNA sequencing rather than whole metagenomic approaches, and many are constrained by limited sequencing depth. To gain a more comprehensive understanding of the gut microbiome's role in athletes, future research should prioritize higher-resolution techniques. Whole metagenomic sequencing, combined with deeper sequencing efforts, will be instrumental in addressing currently understudied or ambiguous areas.

Furthermore, integrating metagenomics with other omics technologies, such as metabolomics and metatranscriptomics, holds great promise. Metabolomics will provide insights into the biochemical pathways and metabolic interactions within the gut microbiome, while metatranscriptomics will shed light on the active processes. These combined approaches, along with longitudinal study formats, will offer a more dynamic and detailed exploration of how the gut microbiome influences athletic performance and health over time.
